# An overview of anoxygenic phototrophic bacteria and their applications in environmental biotechnology for sustainable Resource recovery

**DOI:** 10.1016/j.btre.2020.e00563

**Published:** 2020-11-19

**Authors:** Drishya M. George, Annette S. Vincent, Hamish R. Mackey

**Affiliations:** a College of Health and Life Sciences, Hamad Bin Khalifa University, Qatar Foundation, Doha, Qatar; b Biological Sciences Program, Carnegie Mellon University in Qatar, Qatar; c Division of Sustainable Development, College of Science and Engineering, Hamad Bin Khalifa University, Qatar Foundation, Doha, Qatar

**Keywords:** ALA, 5-Aminolevulinic acid, APB, Anoxygenic phototrophic bacteria, BChl, Bacteriochlorophyll, BES, Bioelectrochemical systems, BPh, Bacteriopheophytin, BPV, Biophotovoltaic, Chl, Chlorophyll, CoQ10, Coenzyme Q10, DET, Direct electron transfer, DO, Dissolved oxygen, DNA, Deoxyribonucleic acid, DXP, 1 deoxy-d-xylulose 5-phosphate, Fe-S, Iron-Sulfur, FPP, Farnesyl pyrophosphate, GNSB, Green non sulfur bacteria, GSB, Green sulfur bacteria, LED, light emitting diode, LH2, light-harvesting component II, MFC, Microbial fuel cell, MVA, Mevalonate, PHA, Poly-β-hydroxyalkanoates, PH3B, Poly-3-hydroxybutyrate, PHB, Poly-β-hydroxybutyrate, Pheo-Q, Pheophytin-Quinone, Photo-MFC, Photo microbial fuel cell, Photo-BES, Photosynthetic bioelectrochemical systems, PNSB, Purple non sulfur bacteria, PPB, Purple phototrophic bacteria, PSB, Purple sulfur bacteria, RuBisCO, Ribulose-1,5-biphosphate carboxylase/oxygenase, SCP, Single-cell protein, SOB, Sulfide oxidizing bacteria, SRB, Sulfate reducing bacteria, IPP, Isopentenyl pyrophosphate isomerase, Anoxygenic phototrophic bacteria (APB), Resource recovery, Purple phototrophic bacteria (PPB), Bacteriochlorophyll (BChl), Poly-β-hydroxyalkanoates (PHA), Single-cell proteins (SCP)

## Abstract

Anoxygenic phototrophic bacteria (APB) are a phylogenetically diverse group of organisms that can harness solar energy for their growth and metabolism. These bacteria vary broadly in terms of their metabolism as well as the composition of their photosynthetic apparatus. Unlike oxygenic phototrophic bacteria such as algae and cyanobacteria, APB can use both organic and inorganic electron donors for light-dependent fixation of carbon dioxide without generating oxygen. Their versatile metabolism, ability to adapt in extreme conditions, low maintenance cost and high biomass yield make APB ideal for wastewater treatment, resource recovery and in the production of high value substances. This review highlights the advantages of APB over algae and cyanobacteria, and their applications in photo-bioelectrochemical systems, production of poly-β-hydroxyalkanoates, single-cell protein, biofertilizers and pigments. The ecology of ABP, their distinguishing factors, various physiochemical parameters governing the production of high-value substances and future directions of APB utilization are also discussed.

## Highlights

• APB are phylogenetically and metabolically diverse, includes many extremophiles.
• Ability to harvest IR light and use of many inorganic electron donors useful.
• Electricity, polymers, fertilizers, feed, antioxidants and pigments recoverable.
• Purple non-sulfur bacteria well studied, have wide range of applications.
• Metabolic engineering key to improving productivity and metabolic traits.

## 1 Introduction

Global warming, ozone depletion, climate change, air and water pollution and lack of proper waste management have all contributed to an acceleration in environmental biotechnology research [1]. Incidentally, this aligns with a number of the 2015 United Nations Sustainable Development Goals that stressed on manufacturing products in an environmentally sustainable and economical manner [2]. Using microorganisms as biocatalysts for the production of biofuels, bioenergy, biodegradable plastics, polymers and bioremediation can advance sustainable development with minimal impact on the environment [1].

Anoxygenic phototrophic bacteria (APB) are a diverse phylogenetic group of bacteria that perform anoxygenic photosynthesis using a variety of organic/inorganic electron donors. They have several desirable characteristics such as: anaerobic growth, low energy requirements, diverse modes of metabolism, low growth and maintenance cost that can be exploited for several environmental biotechnology applications. They can also be utilized to produce a wide range of metabolic and cellular products of value. However, when compared to algae and cyanobacteria, APB based applications have received considerably less attention. This is despite the fact that several life cycle assessment studies have highlighted the unsustainability of algae-derived bioenergy [[3], [4], [5]] and the possible risks in mixed algal cultures of unwanted cyanotoxin production [6]. Therefore, a paradigm shift with a focus on identifying and developing techniques of harvesting bioenergy and value-added substances from APB, is warranted.

There is a growing trend towards integrated wastewater treatment and resource recovery. Resource recovery from wastewater is crucial from both an economical and environmental aspect. Many studies have highlighted the robust and versatile metabolic pathways utilized by APB, making them ideal for resource recovery applications [7,8]. Garcia et al. [9], compared the wastewater treatment abilities of algae-bacteria vs purple phototrophic bacteria (PPB) in treating piggery wastewater in open bioreactors. They demonstrated the ability of PPB to grow in toxic conditions while retaining high organic carbon removal rates, thus making them ideal for outdoor applications when compared to the algae-bacteria consortia.

In order to improve this technology, it is important to identify, characterize and study APB that can be utilized in various industries including: agriculture, aquaculture, pharmaceutical, chemicals and in the production of high value substances. Among the many APB based applications, the production of biohydrogen has very strong potential, particularly given the increased focus on hydrogen as a key future energy carrier and fuel. Recent reviews, including the review by Sampath et al. [10] describes the different biological methods as well as suitable substrates that can be employed for the production of biohydrogen. Tian et al. [11] reviews three technologies that can be used to convert waste matter to biohydrogen with emphasis on both the technological as well as the environmental outlook. Tiang et al. [12] mainly focuses on optimization of photo-fermentative methods for the enhancement of biohydrogen production using PNSB. Given the breadth of recent and comprehensive reviews already available on biohydrogen production, this is one resource that will not be covered in this review. This review will address three major components - a general overview of APB, the potential resources that can be recovered or generated using these organism and how these organisms can be manipulated at a molecular level in order to enhance desirable characteristics. The limitations and future directions of APB based technologies will also be discussed.

## 2 Anoxygenic phototrophic bacteria

Photosynthetic bacteria can be either oxygenic or anoxygenic. They differ based on the type of chlorophyll and carotenoid pigments present, photosynthetic electron donor used as and the composition of their photosynthetic machinery. Anoxygenic phototrophic bacteria can perform photosynthesis without the evolution of oxygen. These bacteria can grow in anaerobic conditions; and for most groups of anoxygenic phototrophs, the presence of oxygen hinders the formation and functioning of their photosynthetic machinery and pigments [13].

### 2.1 Phylogeny and types of anoxygenic phototrophic bacteria

APB are a phylogenetically and photosynthetically diverse group of organisms that share two common traits distinguishing them from the more conserved oxygenic phototrophic bacteria. The first is that they rely on bacteriochlorophylls rather than chlorophyll as the primary photopigment; and the second is that they do not oxidize water, but rather use sulfide, hydrogen, organics or similar electron donors as reducing power for photosynthesis [13,14]. Bacteria belonging to ABP use either type I reaction center or type II reaction center for photosynthesis. A type I reaction center is present in organisms of the phyla *Chlorobi*, *Firmicutes* and *Acidobacteria*; while a type II reaction center is present in organisms belonging to the phyla *Chloroflexi*, *Proteobacteria* and *Gemmatimonadetes* [15]. APB harvest light between 740–1020 nm; therefore, multiple anoxygenic phototrophs can co-exist in the same environment [16,17]. The bacteria, *Chloroflexus aurantiacus,* have large amounts of bacteriochlorophyll *c,* and were initially classified by Oyaizu et al. [18] under a distinct lineage called “green non-sulfur bacteria”; along with the closely related filamentous chemotroph, *Herpetosiphon aurantiacus,* and a non-motile chemotroph, *Thermomicrobium roseum* [19]. However, since these bacteria differed from green sulfur bacteria, both phylogenetically and physiologically, they are currently classified as filamentous anoxygenic phototrophic bacteria [19].

A diverse group of APB called aerobic anoxygenic are obligate heterotrophs, and use bacteriochlorophyll (BChl) *a* as their primary light-harvesting pigment. Unlike APB, this group of bacteria require oxygen for their growth and photosynthetic electron transfer; and they lack Ribulose-1,5-biphosphate carboxylase/oxygenase (RuBisCO) [20,21], an important photosynthetic enzyme that enables the fixation of atmospheric carbon dioxide into carbon-rich organic compounds. Since these organisms are quite distinct from APB and are not well studied for their applications, they will not be explored in this review. Table. 1. lists the various groups of APB and highlights their diverse metabolic features and habitats.

**Table 1:** Distinguishing features of the various types of anoxygenic phototrophic bacteria.

GROUP	ORGANISM	REACTION CENTRE	PHOTOSYNTHETIC PIGMENTS	ABSORPTION WAVELENGTH	ELECTRON DONOR	TYPE OF METABOLISM	ENVIRONMENTS INHABITED	SPECIAL CHARACTERISTICS
*Purple sulfur bacteria*	Belong to the *Gammaproteobacteria* class and classified under *Chromatiaceae* and the *Ectothiorhodospiraceae* families [13]	Type II [22,23]	BChl *a/b* and carotenoid pigments such as *spirilloxanthin, spheroidene, lycopene,* and *rhodopsin* [22,24]	For bacteriochlorophyll *a* ∼ 800/ 815−960 nm. and bacteriochlorophyll *b* containing species have a range of 835−850 and 1010−1040 nm [23]	Inorganic sulfur compounds like sulfide, hydrogen and thiosulfate for photoautotrophic growth [22,24]	Mainly photoautotroph. *Chromatiaceae* can grow well under photoautotrophic, photoheterotrophic, chemoautotrophic and chemoheterotrophic conditions [13]	Found in alkaline and saline environments [13]	During their growth on thiosulfate, elemental sulfur or polysulfides, bacteria from the *Chromatiaceae* family can form sulfur globules within their cell wall whereas organisms that are a part of the *Ectothiorhodospiraceae* family accumulate sulfur globules extracellularly [25]
*Purple non-sulfur bacteria*	Belong to the *Alpha* and *Betaproreobacteria* class	Type II [22,24]	BChl *a/b* and carotenoid pigments such as *spirilloxanthin, spheroidene, lycopene, and rhodopsin* [22,23,24]	For bacteriochlorophyll *a* ∼ 800/ 815−960 nm. Whereas, bacteriochlorophyll *b* containing species have a range of 835−850 and 1010−1040 nm [23]	Photoautotrophic species use reduced sulfur and hydrogen compounds [13]	Mainly photoheterotrophs [22,24]. However, some species can grow photoautotrophically [13]	Thrive in conditions having low sulfide concentrations and can be found in sewage and waste lagoons [26,27]	Can grow in environments with low sulfide levels and during photoautotrophic growth, the reductive pentose phosphate cycle (Calvin cycle) is the path used for CO_2_ fixation [23] S0 formed by oxidation of sulfide is deposited extracellularly [28]
*Green sulfur bacteria*	These bacteria are from the *Chlorobiaceae* family [13]	Type I [22,29]	BChl *c, d* and *e* organized into chlorosomes (large light-harvesting organelles) [22]. Carotenoid pigments such as chlorobactene, *γ*-carotene isorenieratene and derivatives (OH- chlorobactene and *β*-isorenieratene) [30,31]	Bchl *c* 745–755 nm, Bchl *d* 715–745 nm and Bchl *e* 710–725 nm [22,29]	Reduced sulfur compounds such as sulfide and thiosulfate [13]	Obligate phototrophs that grow only in anoxygenic conditions and in the light by utilizing inorganic electron donors [13]	Exist as colored blooms in hyper saline water, can be found in marine lagoons, hypersaline sediments, freshwater lakes and even in marine sediments [32,33]	They play an important role in the biological sulfur cycle and deposit sulfur globules extracellularly; and use the reversed tricarboxylic acid (TCA) cycle to fix carbon dioxide [29].
*Filamentous anoxygenic phototrophic bacteria*	Chloroflexi phylum consists of primarily filamentous organisms and consist of the following three families: *Chloroflexaceae, Oscillochloridaceae* and *Roseiflexaceae* [19]	Type II [22]	BChl *a* or *a* and *c/d* [19]. Different carotenoid pigments such as carotene, β-carotene, OH-*γ*-caroteneglucoside ester, keto-OH-*γ*- carotene, keto- myxocoxanthin, myxobactene, methoxy-keto-myxocoxanthin, keto-myxocoxanthin glycoside ester are present depending on the family [34,35,36]	720−878 nm [34,35,36]	Sulfide or hydrogen used as electron donors [37]	Bacteria belonging to *Chloroflexaceae* family are mainly photoheterotrophs (aerobic conditions) whereas those belonging to *Oscillochloridaceae* are photolithoautotrophs or photolithoheterotrophs (anaerobic conditions) and bacteria from the *Roseiflexaceae* family are facultative phototrophs [13,19]	These bacteria can be found in marine and freshwater mesophilic environments [38] and particularly in microbial mats found in hot springs [39]	They exists as multicellular filamentous organisms in nature and have gliding motility [38].
*Heliobacteria*	These bacteria are classified under phylum *Firmicutes* and *Heliobacteriaceae* family [40]	Type I [22,29]	BChl *g* [13]. Carotenoid pigments such as 4,4’ -diaponeurosporene, OH-diaponeurosporene glucoside esters [41,42]	Optimum absorption is in the range of 786−792 nm [13]	Sulfate utilized by *H. chlorum* [40]	Photoheterotrophs that require light for their growth and depend on organic compounds as their carbon source [42]	Mainly thrive in agricultural and garden soils while only a few species survive in aquatic environments [42,43]	Some species that are found in agricultural soil exhibit a symbiotic relationship with rice plants where the plants act as a source of organic carbon for the bacteria [42,43]
*Acidobacteria*	Phylum *Acidobacteria i*ncludes three families namely, *Acidobacteriaceae*, *Holophagaceae* and *Acanthopleuribacteraceae* [44]	Type I [45]	BChl *c*, BChl *aP*, Chl a*PD* and Zn-BChl *a’P* [46]. Carotenoid pigments such as Echinenone, canthaxanthin, lycopene, *γ* and *β* -carotene may be present [46]	Absorbs infrared light lying between 740–750 nm [47]	They use different sugars and organic compound such as acetate, succinate and propionate for their growth [44]	Mainly chemo-organotrophs but some species are photoheterotrophs [44]	Can be found in hot springs, metal contaminated soils and in marine sediments [44]	These bacteria mainly grow in aerobic environments and some grow under microaerobic conditions [45]
*Gemmatimonadetes*	*Gemmatimonadaceae family* [48]	Type II [49]	BChl *a* [49]	Absorption at 816 and 866 nm by BChl *a* [49,50]	Sulfide and thiosulfate utilized by some *Gemmatimonas spp.* [49,50]	Heterotrophs that can grow aerobically [48]	Found in soil [51] and fresh water lakes in arid conditions [49]	These aerobic organisms can accumulate polyphosphates within their cell [48]

### 2.2 Electron transport chain in anoxygenic phototrophic bacteria

**Fig. 1:**
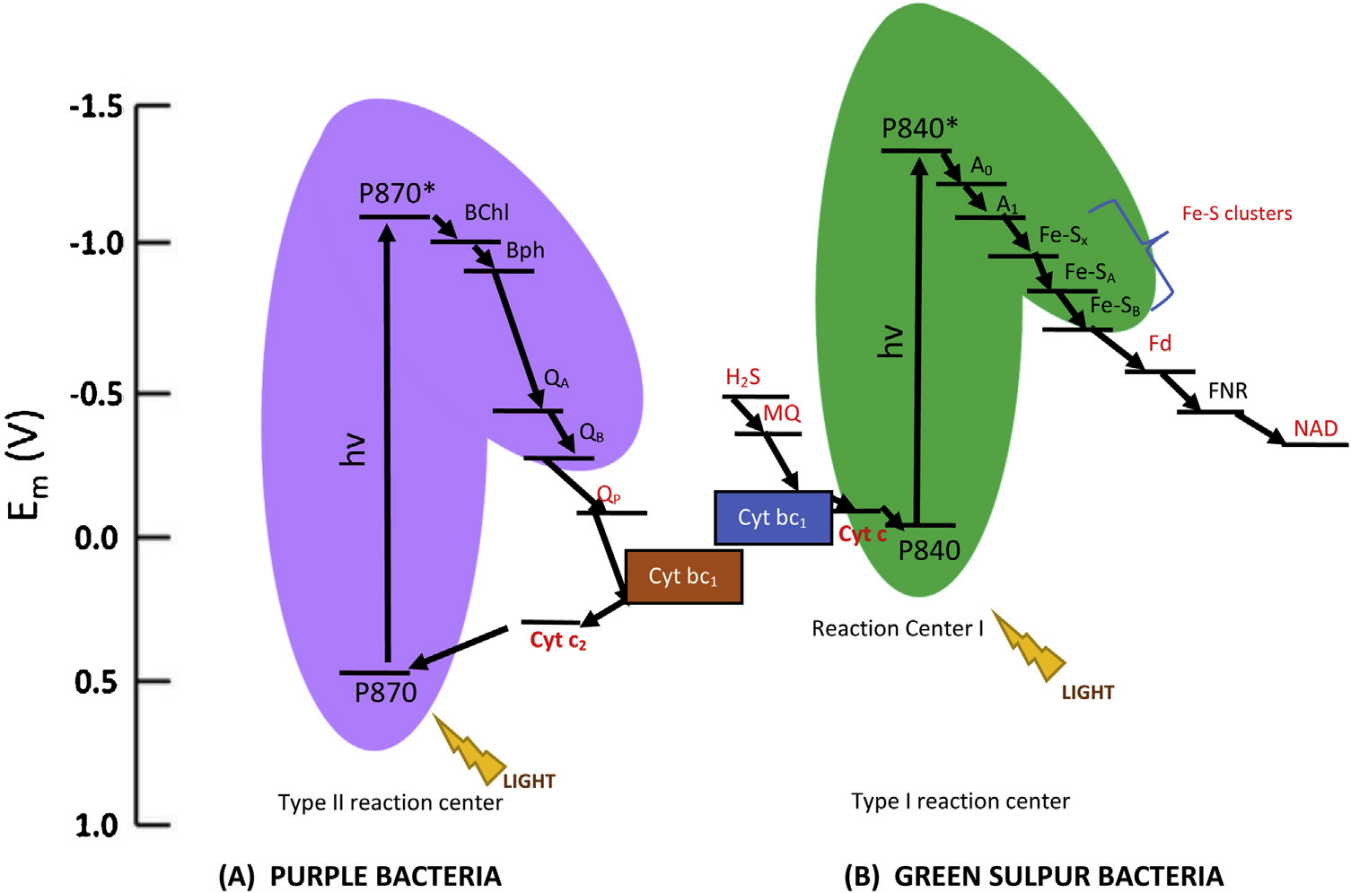
Diagram comparing the electron transport chains in purple bacteria and green sulfur bacteria adapted from Martin Rasmussen and Minteer [53] with (A) Type II reaction center present in purple bacteria and (B) Type I reaction center present in green sulfur bacteria. Pigment 870 (P870), bacteriochlorophyll (BChl), bacteriopheophytin (BPh), primary ubiquinone acceptor (Q_A_), secondary ubiquinone acceptor (Q_B_), ubiquinone pool (Q_P_), cytochrome *bc_1_* (Cyt bc_1_), cytochrome *c_2_* (Cyt c_2_), hydrogen sulfide (H_2_S), menaquinone (MQ), cytochrome *c* (Cyt c), pigment 840 (P840), chlorophyll *a* (A_0_), phylloquinone (A_1_), membrane bound iron sulfide (Fe-S) proteins (Fe-S_x_, Fe-S_A_ and Fe-S_B_), ferredoxin (Fd), ferredoxin-NADP+ reductase (FNR) and nicotinamide adenine dinucleotide (NAD)

Anoxygenic phototropic bacteria such as purple bacteria, have a type II (Pheo-Q type) reaction center [52]. In these organisms, when light energy (in photons) is captured by the light harvesting complexes it is transferred to a dimer of bacteriochlorophyll molecules, pigment P870 (P870), known as the “special pair”. Once the special pair has reached an excitation state (after absorption of light at a wavelength of 870 nm), the electron is transferred to bacteriochlorophyll (BChl), which is the primary electron acceptor. From here the electron is relayed to bacteriopheophytin (BPh) and then to the primary ubiquinone acceptor (Q_A_) [53]. The electron is then passed on to the secondary ubiquinone acceptor (Q_B_), which then accepts two such electrons and two protons from the cytoplasm and moves to the ubiquinone pool (Q_P_). The reduced Q_B_ then gets re-oxidized by cytochrome *bc_1_* complex and releases its protons to the exterior of the cell [53]. An electron from cytochrome *bc_1_* is then passed to cytochrome *c_2_*, which then re-oxidizes the BPh ‘special pair’, thus completing the cycle [53]. The final outcome of the two photons absorbed by the reaction center results in the extracellular transfer of four protons [54]. While the photosynthetic reactions in GSB are similar to those in purple bacteria, the key differences are the absorption wavelength of the BPh special pair (840 nm) and the type of reaction center present in these bacteria. Green sulfur bacteria (GSB) have a type I (Fe-S type) reaction center [52]. The primary electron acceptors in GSB are chlorophyll *a* (A_0_) and phylloquinone (A_1_). Instead of quinones, these bacteria utilize the Fe-S proteins; Fe-S_x_, Fe-S_A_ & Fe-S_B_, to transfer electrons via ferredoxin (Fd) and ferredoxin-NADP + reductase (FNR) to nicotinamide adenine dinucleotide (NAD) [55]. Fig. 1. is an illustration of the electron transport chain present in purple bacteria and green sulfur bacteria.

### 2.3 Niche habitats

#### 2.3.1 Extremophiles

APB inhabit a diverse range of environments and include a considerable group of extremophiles that thrive in habitats having extreme pH, salinity and light. Thermophiles generally inhabit hot springs with temperatures >50 °C, psychrophiles inhabit mats that are found in very cold temperatures of 0−25 °C, and halophiles are found in marine sediments and salterns with high concentration of NaCl [56]. While alkaliphiles thrive in soda lakes at pH > 9, acidophiles are found in acidic soils, lakes and sulfur springs with pH 4–6 [56].

Due to the tolerance of extremophilic APB to extreme environmental conditions, these organisms have a clear competitive edge over others for use in resource recovery that is integrated with treatment of specific industrial wastewaters that may be highly saline, acidic, alkali or with elevated temperature. Furthermore, the use of extremophiles could eliminate the requirement for sterilization in industrial biotechnology applications. Several extremophilic APB, and the environments they inhabit are mentioned below.

##### 2.3.1.1 pH

Alkaliphiles, such as the purple sulfur bacteria (PSB) *Ectothiorhodospira haloalkaliphile* and purple non-sulfur bacteria (PNSB) *Rhodobacabogoriensis,* are found in alkaline lakes and grow in pH ranging from 8.5 to 10.5 [57,58] while acidophiles, such as the PNSB *Rhodophila globiformis* and *Rhodoblastus acidophila,* grow at low pH ranging from 4.2 to 5.8 and inhabit acidic sulfur springs, soils and marshes [33,59].

##### 2.3.1.2 Temperature

Green non-sulfur bacteria (GNSB) such as *Chloroflexus aurantiacus* grow optimally between 55 −70 °C and inhabit marine microbial mats and neutral to alkaline hot springs [60]. Similarly, the GNSB *Roseiflexus castenholzii* are found in hot springs and grow between 50−60 °C [36]. Research on psychrophiles that are APB is limited; the PNSB *Rhodoferax antarcticus* is a psychrophile that was isolated from an Antarctic microbial mat, exhibiting growth at a temperature range between 0−25 °C [24].

##### 2.3.1.3 Salinity

PSB such as *Marichromatium purpuratum* can be found in marine sponges and seawater, and can therefore grow in conditions with up to 7% NaCl [61]. The PSB, *Halorhodospira halophila,* inhabits extremely alkaline environments such as soda lakes and can grow in up to 30 % NaCl [57]. PNSB such as *Rhodovibrio salinarum* and *Rhodovibrio sodomensis* are also found in saline environments including salterns and seas, and grow in up to 20 % NaCl [62].

##### 2.3.1.4 Light

Anoxygenic phototrophs like PSB cannot adapt to regions with low light availability [63]. In contrast, GSB requires only 25 % of the light utilized by PSB for growth [64]. Additionally, some GSB can use blue light for photosynthesis and growth [17]. Alternatively there are some GSB that grow in deep-sea hydrothermal vents and utilize infra-red light from geothermal radiation for photosynthesis [65].

Another group of extremophiles known as *Halobacteria,* are archaebacterial halophiles that exist in environments with high salt concentration such as salt lakes, salterns and even in salted fish [66]. Even though they are devoid of chlorophyll and do not belong to APB, *Halobacteria* can still perform light-dependent CO_2_ fixation under microaerophilic conditions [67]. These organisms utilize specialized proteins called bacteriorhodopsin (similar to the retinal protein found in animals and humans) for photophosphorylation and subsequent CO_2_ fixation [67,68]. They also possess a secondary retinal pigment called halorhodopsin, which is a photo-dependent sodium pump that maintains osmotic equilibrium inside their cells [69]. Since *Halobacteria* can thrive in environments with high salt concentrations and temperature, they can be exploited for the production of several industrially important enzymes and compounds as reviewed by Sekar et al. [70], Kumar et al. [71] and Malik et al. [72].

#### 2.3.2 Microbial mats

Under extreme physical and chemical conditions, APB often exist as a part of a microbial mat that may be terrestrial or in shallow aquatic environments. Types of mats that may harbor APB include (1) Hypersaline mats, which are found in areas with high temperature, light intensity and water salinity; (2) Coastal mats that exist in intertidal costal zones characterized by fluctuations in temperature and salinity (3); Psychrophile mats, which are found in extreme polar environments with high solar radiation and nutrient limitations; (4) Oligotrophic mats that are also found in areas with low nutrient availability, especially in conditions where the concentration of organic compounds are low; and (5) Hot spring mats which exist in environments with high temperature and solar radiation [73]. Acid microbial mats are found are found in places with very low pH and high metal toxicity and unlike most microbial mats, phototrophic microorganisms do not typically inhabit these mats [73]. The diversity of organisms present in microbial mats can be attributed to extreme environmental and physio-chemical factors.

**Fig. 2:**
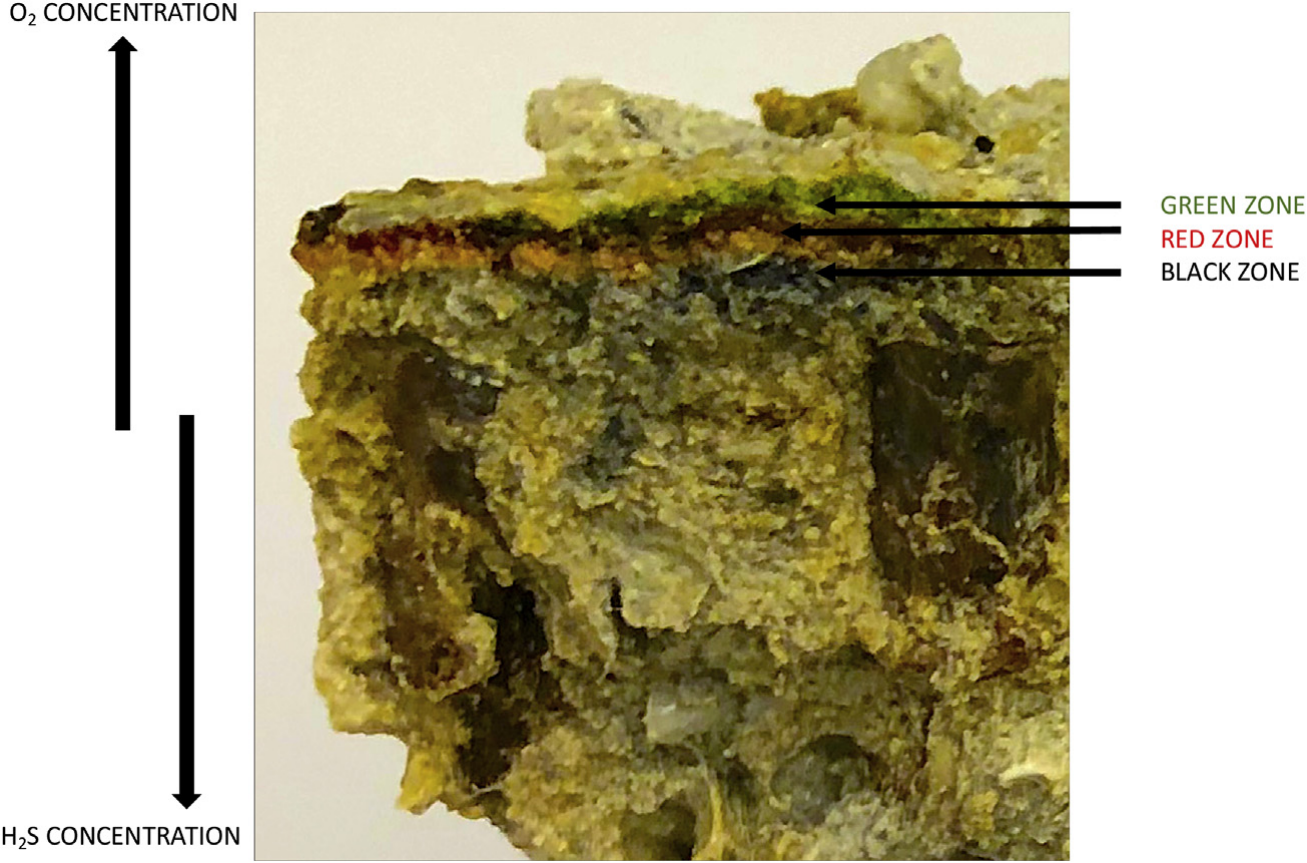
Cross section of microbial mat isolated from Purple Island mangroves, Qatar. Three distinct colored zones can be seen in the mat and are specific to the groups of organisms inhabiting them. Cyanobacteria and algae (oxygenic phototrophs) can be found in the green zone where oxygen concentrations are high and sulfide concentrations are very low. The red zone is inhabited by anoxygenic phototrophs while the black zone, with very low oxygen concentrations and high sulfide concentrations is dominated by sulfate reducing bacteria.

A microbial mat is a complex microbial system. In those driven by photosynthesis, oxygenic phototrophs such as cyanobacteria and algae are usually found at the top layers. These organisms fix inorganic carbon (CO_2_) into organic carbon through the process of photosynthesis [74]. Anoxygenic phototrophs such as GSB, GNSB, PSB and PNSB grow in distinct layers below cyanobacteria [75] while sulfate reducing bacteria (SRB) are confined to anaerobic layers found towards the bottom of the mat [74]. The organic reserves produced by cyanobacteria are broken down by SRB (a chemotrophic organism) which leads to the formation of varying sulfide and oxygen gradients throughout the mat. These gradients influence organisms with different metabolic capabilities and results in their confinement to particular layers within the mat [76] as shown in Fig. 2. *Cyanobacteria* are ubiquitous to all phototrophic microbial mats and, along with ABP belonging to the phyla *Chloroflexi*, *Chlorobi*, *Proteobacteria* (PSB and PNSB) and *Firmicutes,* dominate most phototrophic mats [73].

## 3 Resource recovery applications of anoxygenic phototrophic bacteria

Resource recovery studies utilizing phototrophic bacteria in wastewater treatment have demonstrated the ability of these organisms in nutrient recovery and bioremediation of a wide range of often inhibitory or recalcitrant organics, as well as in the synthesis of value-added products. Their diverse metabolism, absence of associated cyanotoxin production and use of various electron donors make them more versatile than microalgae for phototrophic wastewater and bioremediation applications. The following section details the potential applications of APB for resource recovery.

### 3.1 Photosynthetic bioelectrochemical systems

In bioelectrochemical systems (BES), microorganisms, or a component of them, are used as a biocatalyst at the electrode to facilitate the conversion of electrical energy to chemical energy and vice versa. Compared to conventional fuel cells, BES are an attractive alternative primarily because it utilizes biocatalysts to operate, instead of rare and expensive metal catalysts [77]. Utilizing biocatalysts in BES have several benefits including: lower operational costs, carbon neutrality and reduced resource depletion when compared to standard fuel cells [78]. Generally, BES can operate at a neutral pH [79,80] and ambient temperatures ranging from 15−45 °C [81,82]. Employing extremophiles could extend the operational range of BES considerably, allowing operational temperature ranges beyond that of conventional photovoltaics. BES can be used for the remote storage/generation of energy [83,84], synthesis of biologically derived organic compounds [[85], [86], [87]], as self-powered biosensors [88,89] and for the treatment of wastewater and bioremediation applications [[90], [91], [92]].

**Fig. 3:**
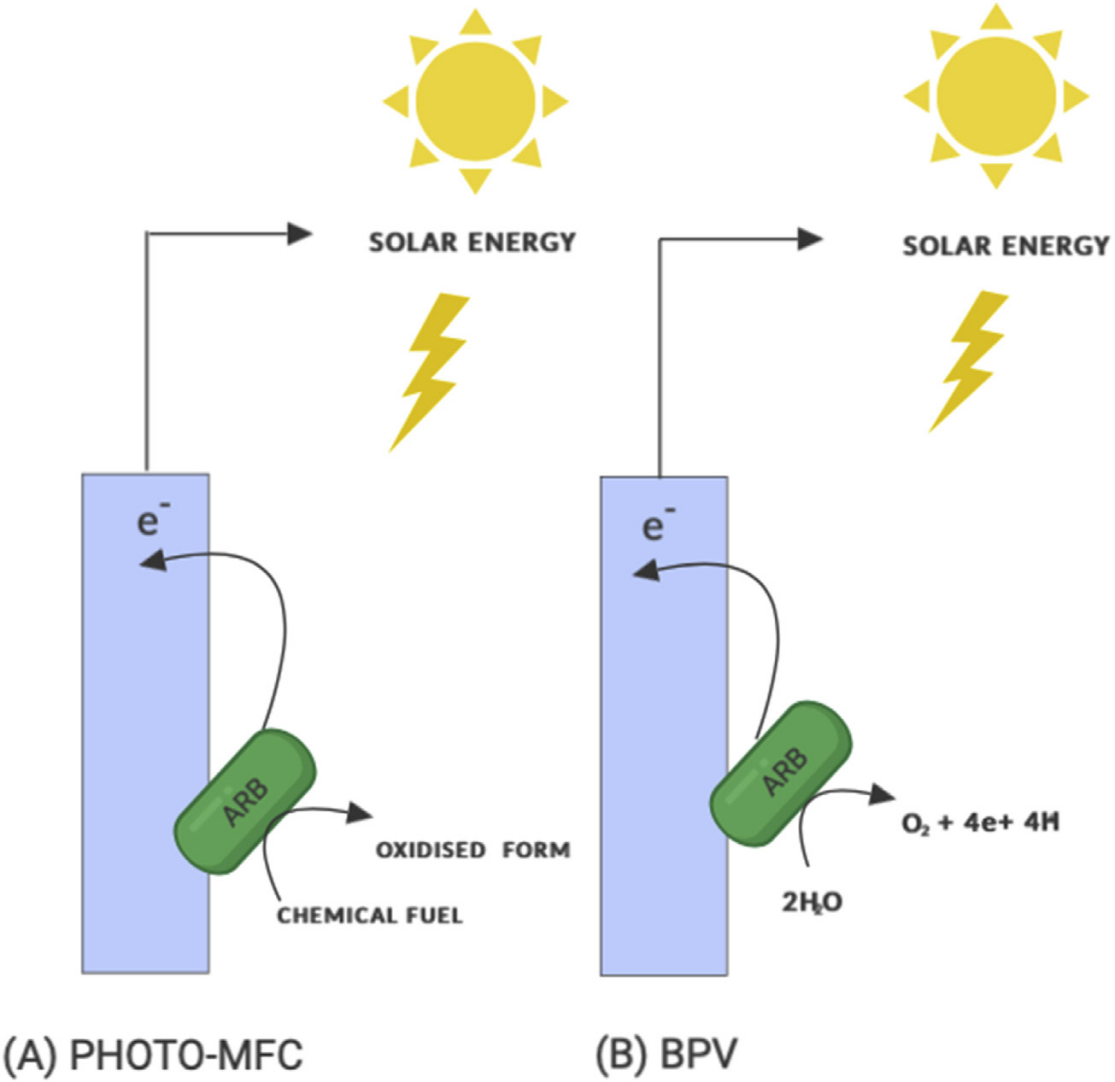
Types of photosynthetic bioelectrochemical systems (A) Photosynthetic microbial fuel cell (Photo-MFC) and (B) Biophotovoltaic system (BPV) with phototrophic anode respiring bacteria (ARB) as the biocatalyst adapted from Jeuken [93].

Photosynthetic bioelectrochemical systems (Photo-BES) harness solar energy and convert it to electrical energy by employing at least one type of photosynthetic microorganism with exoelectrogenic abilities. Biophotovoltaics (BPVs) and photo-microbial fuel cells (Photo-MFC) are types of Photo-BES that utilize phototrophic organisms to harness solar energy and convert this to electrical energy through photosynthesis. However, they differ in the type of phototrophic organism used and their electron source, which can be from either inorganic or organic compounds [93], as shown in Fig. 3.

In BPV, the use of oxygenic exoelectrogens such as certain algae and cyanobacteria in the system results in the evolution of oxygen at the anode during photosynthesis. Oxygen, a well-known electron acceptor, consumes the electrons produced by the organisms, thereby interfering with the total current output produced by the system [94]. On the other hand, using exoelectrogenic APB that utilize reduced inorganic compounds as photosynthetic electron donors prevents oxygen generation in the system [90,95]. Nonetheless, cyanobacteria or algae can still be used in photo-BES as biocathodes, in the place of expensive cathodes made of platinum or platinized carbon [96].

#### 3.1.1 Anoxygenic phototrophic bacteria in photosynthetic bioelectrochemical systems

Exoelectrogenic organisms follow two modes of electron transfer: direct electron transfer (DET) and indirect electron transfer, both of which may occur between two organisms or between an organism and an electrode [93]. By utilizing exoelectrogens capable of DET higher power densities can be generated in BES. This is due to increased mass transfer efficiency between the substrate and electrode [97]. Exoelectrogenic organisms capable of DET can be used to engineer self-sustainable Photo-BES. Where interspecies electron transfer occurs, redox mediators can be cycled and light/dark metabolisms of the different organisms can be potentially harnessed.

Liu and Choi [98] constructed a self-sustaining micro MFC that could generate electric current continuously for 13 days via a syntrophic association between photosynthetic bacteria and heterotrophic exoelectrogens, where the photosynthate produced by the phototroph was utilized as a substrate by the heterotroph. While this particular study was with microalgae, similar potential exists using anoxygenic phototrophs. Ha et al. [99] also demonstrated direct interspecies electron transfer in co-culture experiments that utilized an anoxygenic phototroph *Prosthecochloris aestuarii* (a GSB) and a heterotroph *Geobacter sulfurreducens* They deduced that electron transfer by *G. sulfurreducens* supported the photoautotrophic growth of *P. aestuarii. G. sulfurreducens* oxidized acetate present in the medium to produce carbon dioxide and electrons. This study demonstrated that APB could participate in direct interspecies electron transfer.

Xing et al. [100] reported that a novel strain of *Rhodopseudomonas palustris* strain DX-1 was one of the few PNSB that could generate high power densities (2720 ± 60 mW/m^2^) through direct electron transfer, even without sunlight. *R. palustris*, strain RP2 was utilized by Venkidusamy et al. [91] in bioremediation studies (acetate fed MFC) to degrade hydrocarbon contaminants and the maximum power density produced was 132 ± 10 mW/m^2^. Venkidusamy et al. [101] studied the charge propagation by *R. palustris* RP2 and provided definitive evidence from scanning probe studies that direct electron transfer by this strain was through bacterial nanowires. A recent study by Sun et al. [102] tested the effect of different poised potentials on intracellular electron production from a co-culture of *Chlorella vulgaris* and *R. palustris.* The maximum peak current was generated at a potential of 0 V and was mainly due to the photoheterotrophic metabolism of *R. palustris;* however, *C. vulgaris* required a higher potential and an exogenous electron mediator for electron generation. The metabolic versatility of *R. palustris* allows it to switch between various metabolic modes; from photoheterotrophic to photoorganoautotrophic, chemoorganoheterotrophic, chemoautotrophic and photolithotropic; and can therefore be exploited for a variety of photo-BES based applications [91].

Several APB are also sulfide oxidizing organisms, hence sulfur cycling is possible in these systems, where sulfide is oxidized to donate electrons to the anode and reduced by sulfate reducers. The sulfur cycling can play an important role in photo-MFCs as sulfur is a naturally abundant element whose original redox state can easily be regenerated. This implies that the major disadvantage of photo-MFCs, namely that they require a constant source of fuel, can be resolved by cycling the sulfur species using suitable SRB or SOB at the electrodes.

#### 3.1.2 Factors affecting the performance of photosynthetic bioelectrochemical systems

Several important physiochemical factors such as pH, temperature, salinity and light can influence overall current generation in the Photo-BES by affecting the growth, metabolism and electron generation by APB.

##### 3.1.2.1 pH

Based on the type of application, pH can significantly affect the performance of a BES. In the case of biohydrogen production, the pH has to maintained between 6.5–7 to allow for efficient biohydrogen production [86]. Low pH can also limit electron generation by the anodic biofilm as observed by Lusk et al. [103]. They reported that as the anodic biofilm grows and matures it leads to the accumulation of protons within the biofilm, resulting in the creation of pH gradients. Consequently, the interior of a mature biofilm will have a lower pH, which may limit biofilm growth and electric current production by the organisms.

##### 3.1.2.2 Temperature

Temperature is one of the critical operating factors that can affect overall growth and metabolism of APB and can affect the total current generation in a BES. Wang et al. [104] reported an increase in current density from 3.21 ± 0.2 A/m^3^ to 4.03 ± 0.5 A/m^3^ when the temperature was increased from 20 °C to 50 °C. Cheng et al. [105] inferred from their studies that start-up temperatures of about 30 °C were optimal for the efficient functioning of the MFC while start-up temperatures between 4−10 °C delayed power generation. Therefore stable operating conditions are imperative for the effective functioning of MFCs; and the operating temperature should be close to the actual temperature of the wastewater to be treated [106]. Extremophilic APB can thus be exploited for the generation of electric current and treatment of various effluents of high temperature from the oil and gas industry. Therefore, it is beneficial to identify APB that can exist at temperatures similar to the operational temperature of BES for similar applications.

##### 3.1.2.3 Salinity

High salinity improves overall MFC power production by increasing the conductivity of electron and proton transfer within the BES [107,108]. An increase in conductivity improves proton transfer which consequently reduces the internal resistance of the entire system [107]. Grattieri et al. [109] implemented a salinity adaptation strategy using *Rhodobacter capsulatus.* The authors observed an increase in current from 2.9 ± 0.1–3.2 ± 0.2 μA/cm^2^ for *R. capsulatus* cells that adapted to 15 and 20 g/L NaCl, respectively. Similar Photo-BES using marine APB can be developed to monitor seawater quality and treat highly saline wastewater [109].

##### 3.1.2.4 Light

For Photo-BES, the type and intensity of available light can impact microbial growth and enrichment within the system. In a study conducted by Gürgan et al. [110], they found that in *R. capsulatus* photosynthesis-related genes were downregulated when the organism was exposed to a light intensity of 10,000 lx. However, there was an upregulation of electron transport system genes with increased biohydrogen production to dissipate excess electrons. Although excess light can result in cell damage of phototrophic organisms, it can increase exoelectrogenic activity due to dissipation of reducing power. However, the light saturation peak will differ depending on the APB type. Qi et al. [111,112] demonstrated that APB can grow using weak infrared light emitting diodes (LEDs) at a wavelength of 850 nm, but this same wavelength prevented algal growth. The use of LED lights in Photo-BES are therefore a more economical and effective alternative to incandescent lamps.

### 3.2 Poly-β-hydroxyalkanoates

Poly-β-hydroxyalkanoates (PHA) are ubiquitous microbial biodegradable aliphatic polyesters. They are commonly found in prokaryotic cells as fluid inclusions, encompassed by a protein-phospholipid monolayer [113]. These high molecular weight isotactic biopolyesters are made up of a variety of repeating units that contribute to thermoplastic, elastomeric and biodegradable properties of PHA, thus making it viable for commercial applications [113]. Bacteria produce PHA as a means of storing carbon and other energy reserves during unfavorable conditions, like limited light and availability of nutrients [114]. Apart from acting as an energy reserve, PHA can protect bacteria from extracellular stress by preserving the integrity of the cell; and regulate cell metabolism, morphology and physio-chemical makeup of the organism [115,116]. It also plays a role in the conservation of sulfur cycle and anoxygenic photosynthesis within microbial mats, by acting as an electron and a carbon source respectively [117,118].

Unlike petroleum derived plastics, PHA are environmentally safe and completely biodegradable. These bio-thermoplastics can be used for the production of medical as well as consumer goods [118]. The synthesis of PHA depends on the type of monomer units incorporated, the source of carbon and the metabolic pathways utilized by the organism [113,119,120]. The predominant monomers utilized include 3-hydroxybutyrate (3HB) and 3 -hydroxyvalerate (3 HV). Among anoxygenic phototrophs, PHA was first identified in the purple non-sulfur bacteria - *Rhodospirillum rubrum* [121]. However, apart from PNSB, they are also formed in PSB and aerobic anoxygenic phototrophs [120,122,123].

#### 3.2.1 Biosynthetic pathway of poly-β-hydroxyalkanoates

PHA are categorized into three classes based on the number of carbon atoms present in its monomer units: (i) short-chain length (C3-C5), (ii) medium-chain length (C6-C14) and (iii) long-chain length (>C14) [124]. Higuchi-Takeuchi et al. [120] discovered in a screening test of marine purple bacteria, that while many marine PNSB could produce copolymers of 3HB and 3 HV, some marine PSB could only produce 3HB homopolymers.

To date, over 14 biosynthetic pathways for PHA have been characterized, and acetyl-CoA has been identified as a vital precursor for several short and medium-chain length PHA monomers [125]. The synthesis of poly-β-hydroxybutyrate (PHB), a type of PHA, is not only the first, but is also the most well-studied pathway for PHA biosynthesis. The three key enzymes involved in the synthesis of PHB include: β-ketothiolase (*phbA*), acetoacetyl-CoA reductase (*phbB*) and PHB synthase (*phbC*) [126]. Out of these, PHB synthase is the key enzyme involved in PHB synthesis and based on the type of substrate and the composition of the enzyme subunit, it can be sub-categorized into class I,II,II and IV [127]. Class I PHA synthases produce PHA with high molecular mass (500,000 – several million kDa), class II synthases produce PHA with low molecular mass (50,000–500,000 kDa) and the molecular mass of PHA synthesized by class III PHA synthases are between that of class I and II PHA synthases [131]. The PNSB *Allochromatium vinosum* [128], possess a class III synthase while marine PNSB *Rhodovulum sulfidophilum,* has a class I synthase [127].

**Fig. 4:**
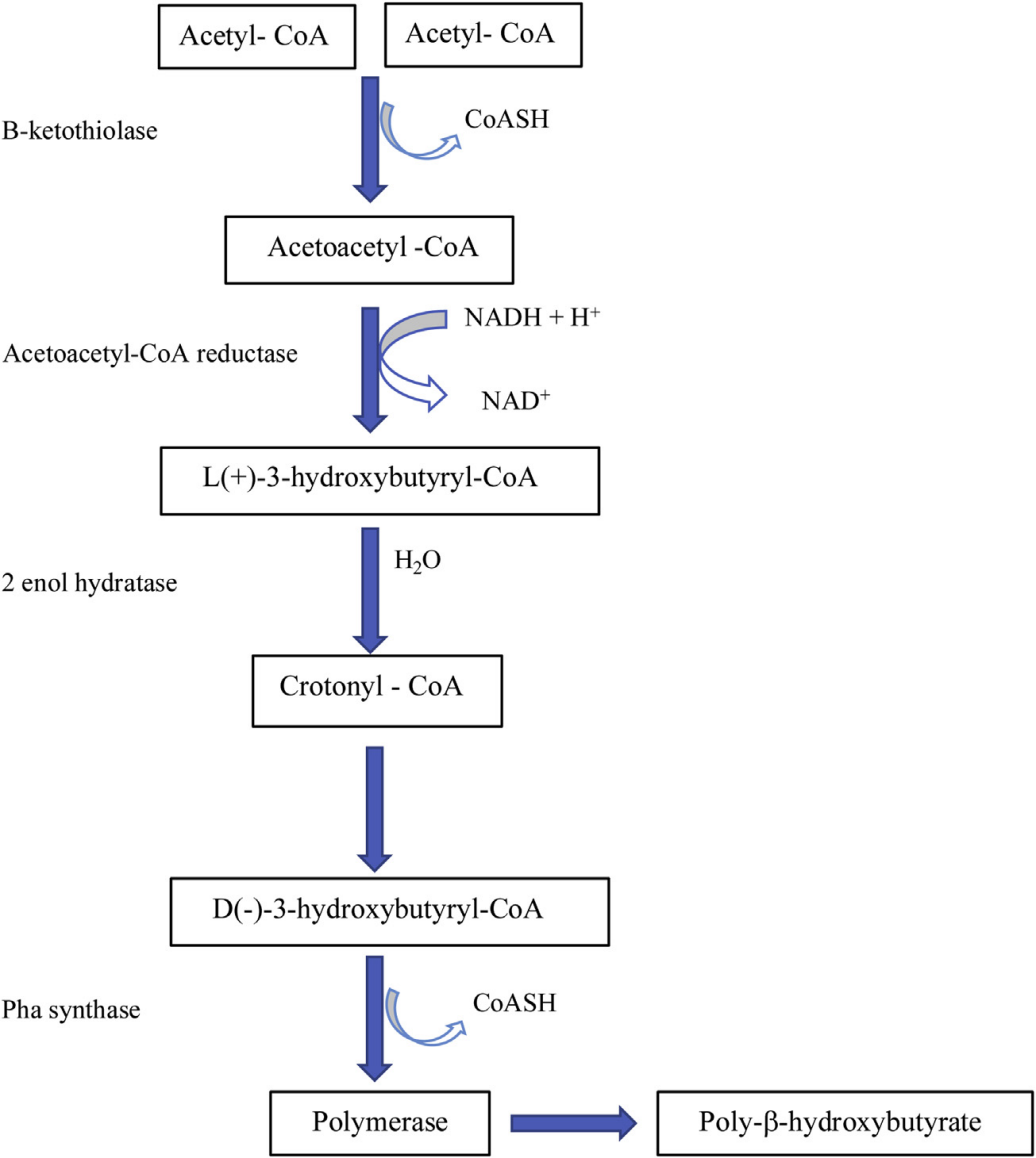
Outline of poly-β-hydroxybutyrate biosynthetic pathway in *R. rubrum* modified from Sagong et al. [124] and Fuller [113].

The synthesis of PHB in *Rhodospirillium rubrum* (PNSB) begins with the condensation of 2 Acetyl-CoA to acetoacetyl-CoA catalyzed by β-ketothiolase [126] as outlined in Fig. 4. Acetoacetyl-CoA reductase then reduces acetoacetyl-CoA to L(+)3-hydroxybutyryl-CoA utilizing nicotinamide adenine dinucleotide phosphate (NADPH) as a co-factor [129,130]. L(+)3-hydroxybutyryl-CoA is converted to the D(-)3-hydroxybutyryl-CoA from by the action of enoyl-CoA hydrolase [129,130]. A decrease in the activity of PHA depolymerase can facilitate the production of high molecular weight PHA with beneficial mechanical properties such as improved tensile strength, an important property required for industrial production [120,132]

The cost of PHA is greatly influenced by the substrate cost. Raw material cost accounts for around 40 % of the total operating cost for PHA production, of which the carbon source accounts for over 70 % [133]. By exploiting carbon rich 2nd generation feedstock such as: waste, glycerol and volatile fatty acids from biodiesel and waste processing industries, PHA production can become more economical [134]. Non-toxic organic substrates like wastewater from the soybean meal, cheese whey, olive mill, sugar, molasses industry [[135], [136], [137]]; as well as carbon-rich lignocellulosic wastes such as spent coffee and waste from the paper and food processing industries [138] have also been studied for PHA production. Padovani et al. [139] have demonstrated the ability of *Rhodopseudomonas* sp. S16-VOGS3 to produce poly-3-hydroxybutyrate (P3HB), a form of PHB, from crude glycerol. Fradinho et al. [140] exploited fermented cheese whey for PHA production, by employing mixed phototrophic bacteria. They found that low concentrations of acetate <30 CmM, and a light intensity of ∼ 20 W/gX, increased PHB accumulation from 15 % up to 30 % in less than 4 h. This was the first study that utilized real waste for PHA production. Studies by Stephanie et al. [141] assessed the use of syngas as a substrate, under nutrient limitations, for PHA production by *R. rubrum*.

Coupling PHA synthesis with wastewater treatment or biohydrogen generation can also lower the overall cost of PHA. Fradinho et al. [142] implemented a novel operational strategy, a permanent feast regime, for the selection of photosynthetic mixed cultures that could produce PHA with high phosphate removal rates. This regime enabled the accumulation of 60 % PHB. Similar strategies can potentially be implemented to integrate wastewater treatment with concurrent PHA production. The co-production of biohydrogen and PHA was studied by Arumugam et al. [143]. They studied PHA production by alternating dark and photofermentation of *Calophyllum inophyllum* oil cake by utilizing *Enterobacter aerogenes* and *Rhodobacter sphaeroides*. The co-production of PHA and biohydrogen was also evaluated by Padovani et al. [144] by growing the PNSB, *Rhodopseudomonas palustris*, on wastewater from an olive mill. However, this also forms a competing use of reducing equivalents, such that conditions must be carefully controlled to minimize biohydrogen production if the main product of interest is PHA.

#### 3.2.2 Factors governing the synthesis of poly-β-hydroxyalkanoates in anoxygenic phototrophic bacteria

Factors such as pH, temperature, salinity, aeration and nutrients can affect PHA accumulation in APB. A neutral to alkaline pH is ideal for PHA synthesis, while an acidic pH leads to competition between hydrogen production and PHA accumulation [145]. Acidic conditions may also lead to inhibition from undissociated volatile fatty acids. While PHA was synthesized by few marine PSB under nitrogen-limited conditions, some marine PNSB did not require nutrient limited conditions for PHA accumulation [120]. Illumination cycles, light intensity and specific wavelengths of light can also affect PHA synthesis. Higuchi et al. [146] observed increased PHA production for cultures enriched under phototrophic conditions at a wavelength of 800 nm compared to those enriched at 730 and 850 nm. Recent studies reporting the effects of the above factors on PHA synthesis by PNSB are summarized in Table 2.

**Table 2:** Factors affecting the accumulation of poly-β-hydroxyalkanoates.

FACTOR	CONDITIONS	AFFECT	MOLECULAR MECHANISM	GROUP	ORGANISM	REFERENCE
Nutrient	No nutrient limitation	PHA 302 mg/L culture	High molecular weight PHA was produced due to decreased activity of PhaC and PhaZ	Marine PNSB	*Rhodovulum visakhapatnamense*	[120]
Nutrient	Addition of 1−2 μm of iron under photoheterotrophic micro-aerobic conditions	PHA accumulation of ∼20−30% cell weight	During log phase, *phaC* and *phaZ* expression were relatively the same. However, during log phase the expression of *phaC1* transcript and *phaZ* increased which consequently led to a decrease in PHA accumulation.	Marine PNSB	*Rhodovulum sulfidophilum*	[147]
Nutrient	Syngas devoid of carbon monoxide and phosphorous, in fed- batch cultivation with controlled acetate input	30 % w/w PHB produced.	CO was converted to carbon dioxide via the action of CO dehydrogenase. Carbon dioxide was then fixed through the Calvin Benson cycle and consequently led to the assimilation of acetate as PHB [148]	PNSB	Rhodospirillum rubrum	[141]
Light	3 wavelengths – 730, 800 and 850 nm. Low light intensity of 8 W/m^2^ and high light intensity of 50 W/m^2^.	A wavelength of 800 nm showed increased PHA production compared to 730 and 850 nm. Low intensities of light allowed for PHA accumulation (17−50 wt%) compared to high light intensities (15−30% wt)	Although there were no significant changes in the expression levels of *IDH* (isocitrate dehydrogenase), PhaC, PhaP and PhaZ, the changes in the TCA cycle and biosynthetic pathway of PHA could not account for low accumulation of PHA under high light intensities. However, a >3-fold elevation in the expression of *PdhR* – (pyruvate dehydrogenase complex regulator) under high light conditions indicated the involvement of the GntR family transcription factor. This was possible via an alternative metabolic pathway in order to protect cells from photooxidation caused due to high light intensities.	Marine PNSB	*Rhodovulum sulfidophilum, Rhodovulum euryhalinum, Rhodovulum imhoffii, and Rhodovulum visakhapatnamense*	[146]
Light	Three illumination cycles (light: dark): 15:15, 30:30 and 60:60 minutes	PHB accumulation highest under 30:30 min light:dark cycles, (308 ± 2 mgPHB/gdw) (24% wt) compared to continuous illumination	Highest PHB accumulation occurred when H_2_ production was the lowest. This was due to the rerouting of protons and electrons towards PHB accumulation instead of it being used for H_2_ production. With regards to the light/ dark - cycling due to the inhibition of nitrogenase activity which is required for H_2_ production in the dark, the activity of the TCA cycle was limited [149] and resulted in increased PHB accumulation.	PNSB	*Rhodobacter capsulatus*	[150]
Oxygen	PHA production under anaerobic vs aerobic conditions	Under anaerobic conditions, PHA content was ∼ 43 % wt. However, under aerobic conditions, the PHA accumulation was only between 0.4−1.8% wt. When acetate was added to the culture, under aerobic conditions, in the case of *R. sulfidophilum* 33% wt PHA was produced	Addition of acetate under aerobic conditions increased *IDH* expression by 10-fold, due to increased activity of the TCA cycle. External addition of acetate to the media resulted in the direct production of acetyl-CoA and an enhancement of the ethylmalonyl-CoA (EMC) pathway [151]. Induction of the EMC pathway (shared PhaA and PhaB enzymes with the PHA pathway) therefore enhanced PHA accumulation.	Marine PNSB	*Rhodovulum sulfidophilum, Rhodovulum imhoffii,* and *Rhodovulum visakhapatnamense*	[146]
pH	pH 7, 8 and 9 tested	At pH 8, P3HB accumulation was highest at 412 ml/L but pH 9 caused a drop in the P3HB accumulation.	As pH increased >7, P3HB accumulation increased. However, as pH increased >8, PHA depolymerase was activated and resulted in the reduction of P3HB.	Marine PNSB	*Rhodovulum sulfidophilum* DSM-1374	[152]
pH	Acetate concentrations – 10, 25, 50 and 65 mM.	At high concentrations of acetate (65 mM), the pH was >7.2 and resultant PHB accumulation was also at its highest- 234.7 mg/L (>20% cdw)	A high acetate (carbon) concentration of 65 mM, led to an increase in the mRNA transcripts in the organism. This resulted in an increase in enzymes involved in PHA biosynthesis, particularly *pha C,* which enhanced PHB production. Low pH (<7), was optimal for H_2_ production. Thus, as the pH increased, H_2_ production decreased significantly and PHB accumulation increased	PNSB	*Rhodobacter capsulatus* DSM 1710	[145]
Temperature	25, 30 and 35 °C	Highest P3HB production was observed at 25 °C - 428 ± 11 mg/L (192 h of cultivation).	BChl concentration was the lowest for cultures grown at 25 °C. Generally, PHA accumulation occurred when cell growth was inhibited. Hence as the BChl concentration decreased, PHA accumulation increased.	Marine PNSB	*Rhodovulum sulfidophilum* DSM-1374	[152]
Salinity	NaCl - 1.5, 2.5, 3.5 and 4.5 %	Highest P3HB obtained when cultures were growth at salinity of 4.5 % (820 ± 50 mg/L) and lowest observed at 1.5 % (388 ± 32 mg/L)	*Rhodovulum sulfidophilum* DSM-1374 is a marine PNSB with a high tolerance to salt. Therefore, highly saline conditions were ideal for its general metabolism and PHA accumulation.	Marine PNSB	*Rhodovulum sulfidophilum* DSM-1374	[152]

### 3.3 Biofertilizers

Biofertilizers are composed of living microorganisms that can be applied directly to the plant or the soil to improve soil fertility and enhance plant growth. Unlike conventional fertilizers; biofertilizers are non-toxic, organic and can improve plant growth while simultaneously conferring them with resistance against certain diseases [153,154]. When compared to cyanobacteria several characteristics of APB including: their ability to grow under subsurface soils [155], faster growth rates, ability to utilize near and far infra-red light for growth, low culture and maintenance cost, ability to fix nitrogen in the dark anaerobically [156] and the absence of production of toxic compounds [6] make them suitable for use as biofertilizers.

APB have intracellular phosphate reserves that can increase the availability of phosphorous in the soil [157]. These bacteria can also enhance soil enzyme activity [158], fix nitrogen via in vivo nitrogenase activity [159,160] and generate useful organic compounds such as amino acids, organic acids and nucleic acids that are vital for plant growth [161,162]. PNSB increase the abundance of copiotrophic bacteria from the phylum Firmicutes that act as biocontrol agents against pathogens [154]. Su et al. [153] studied the use of *R. palustris* G*J*-22 spray in protecting the tobacco plant against the tobacco mosaic virus. APB based biofertilizers can either be applied directly to the soil to boost fertility [163] or be used as a foliar spray [164]. Wu et al. [165] reported improved growth of *Stevia rebaudiana* when a *Rhodopseudomonas* sp. (ISP-1) based foliar spray was used. While direct application to the soil stimulated the metabolism of soil bacteria which consequently improved plant growth, the use of a foliar spray improved the activity of phyllosphere microorganisms that led to enhanced photosynthetic activity and stevioside yield.

Apart from improving plant growth, PSB and PNSB have been previously used in soil bioremediation studies. Habte and Alexander [166] reported the ability of APB to detoxify soil containing high amounts of hydrogen sulfide, by converting sulfide to sulfate via the sulfur cycle. Balasubramanya and Patil [167] reported the ability of *Rhodospirillum* sp. in the degradation of fungicides such as carboxin and oxycarboxin. Although certain herbicides like propanil (3′,4′-dichloropropinonanilide) impeded the growth and nitrogenase activity of cyanobacteria, it led to an increase in soil nitrogenase activity of PSB such as *Thiospirillum* and *Chromatium*. Sakpirom et al. [163] deduced that PNSB were able to reduce methane emissions as well as cadmium and zinc present in a contaminated soil and could therefore be used for bioremediation purposes. Similar bioremediation studies using PNSB were conducted by Wu et al. [158] for the removal of imidacloprid from soil and mitigation of tetrabromobisphenol-A (TBBPA) induced toxicity in soybean seedlings.

#### 3.3.1 5-Aminolevulinic acid

5-Aminolevulinic acid (ALA), or 5-amino-4-oxo- pentanoic acid, is the aliphatic precursor for tetrapyrrole synthesis, and is known to be an effective herbicide and insecticide [168]. It is a photodynamic compound also found in algae, plants and animals, that is capable of converting molecular oxygen into singlet oxygen upon excitation by light [168]. As reported by Rebeiz and Hopen [169], excitation of singlet oxygen by light causes the oxidation and damage of phospholipid membrane and consequently results in plant death. Because the compound is more effective on dicots (seeds with two leaves) than monocots (seeds with one leaf) it allows selective targeting of weeds, which are typically dicots when used with monocots such as grass and many cereals. While this compound is produced only in small quantities by plants and animals, large amounts are produced by microbes.

Synthesis of ALA in PNSB follows the C_4_ pathway where 5-aminolevulinic acid synthetase also known as ALA synthetase condenses succinyl-CoA and glycine to form ALA [170]. Among known APB, the PNSB *R. palustris* [171] and *Rhodobacter sphaeroides* [172]; and the GSB *Chlobiium limicola* and GNSB *Chloroflexus aurantiacus* produce ALA [171]. PNSB have been widely studied for its bioremediation capabilities and application as a biofertilizer; some recent studies are summarized in Table 3.

**Table 3:** Bioremediation and biofertilizers studies utilizing purple non-sulfur bacteria.

ORGANISM	CONDITIONS	TESTED ON	EFFECT	PARAMETERS	REFERENCE
*Rhodobacter capsulatus*	Cultured in bioreactors under dark aerobic conditions. Soybean processing wastewater was used as a substrate. Five treatment groups: 1000 mg/L *R. capsulata* with water and no effluent (control), 500, 1000, 1500 and 2000 mg/L *R. capsulata*	Silt loam soil	The imidacloprid removal rate was the highest in the 1000 mg/l dose group. Improved soil fertility was also reported.	Increased soil urease, catalase and sucrase activity. There was also an increase in soil organic matter and soil microbial carbon content.	[158]
*Rhodopseudomonas sp.,*	Indoor pig farm wastewater was used as the growth substrate. Cultivation was done in flat plate reactors (allow the formation of attached biofilm)	Common pasture ryegrass (*Lolium rigidum Gaudin*)	Improved soil fertility	When the soil was treated with PPB biomass, this increased the abundance of bacteria belonging to the phylum *Firmicutes*. Several organisms belonging to this phylum are known to promote plant health and growth by protecting them from pathogens	[154]
*Rhodopseudomonas palustris* GJ-22	Bacteria were cultivated in liquid cultures under photo-anaerobic conditions	*Nicotiana tabacum* (tobacco plant)	Foliar spray of GJ-22 suspension protected the tobacco plant against tobacco mosaic virus. It also promoted the growth and germination of the plant	*R. palustris* GJ-22 colonized the plant phyllosphere and it produced phytohormones 5-aminolevulinic acid and indole-3-acetic acid which conferred the tobacco plant with resistance against the virus. Upregulation of the plant defense genes -*PR* and *RDR* were also reported.	[153]
*Rhodopseudomonas palustris* TN110 and *Rubrivivax gelatinosus* TN414	Isolated soil samples were cultured anaerobically in the presence of light	Soil, sediment and water samples contaminated with cadmium and zinc, isolated from paddy fields in Thailand	These bacteria produced ammonium, and plant growth promoting substances – ALA and indole-3-acetic acid (IAA). It reduced methane emissions, cadmium and zinc present in the contaminated soil.	Strain TN110 produced the highest concentration NH_4_ - 3.20 ± 0.33 mg/L, ALA - 4.32 ± 0.10 mg/L and IAA - 3.62 ± 0.26 mg/L. Around 84% of cadmium and 55% of zinc were degraded by this strain.	[163]
*Rhodopseudomonas palustris*	Anaerobically cultured with light in Stevia residue medium with different N sources - l-tryptophan and NH_4_Cl.	Chinese pak choi cabbage (*B. chinensis L*.) Foliar spray of *R. palustris* with L-tryptophan (ExT) and with NH_4_Cl (ExN)	There was an increase in biomass and the high net rate of plant leaf photosynthesis when ExT foliar spray and ExN were used. An increase in the abundance of bacteria known to stimulate plant growth, act as biocontrol agents and take part in carbon cycling and nitrogen fixation was also reported.	Net photosynthetic rate and biomass were highest for the ExN foliar spray, 14.31 μmol CO_2_/m^2^/s and 39.40 g/pot. Increase in photosynthetic rate could be due to the production of ALA, a precursor to chlorophyll synthesis [173]. There was also an increase in the abundance of *Acidobacteria*, *Actinobacteria*, *Proteobacteria*, *Gemmatimonadaetes*, *Nitrospirae*, and *Planctomycetes* that were known to protect and improve plant growth.	[164]
*Rhodobacter sphaeroides*	PNSB isolated from oil field injection water with optimum temperature between 30−35 °C; pH of 7 and inoculum size of 2 X 108 m/L.	Wheat seedling and soil samples from a test field.	Change in the speciation of lead (Pb) in the contaminated soil samples to a more inert phase. For the wheat seedling experiment, there was a reduction in the phytoavailability of lead in the contaminated soils.	Bioremediation efficiency on the root and leaf of wheat was 14.78 % and 24.01 % respectively. Although there was no change in the total content of lead in the contaminated soil, it was hypothesized that the mechanism by which *R. sphaeroides* acted on the contaminated soil was by converting the lead to inert compounds such as lead sulfate and lead sulfide. As a result, there was a reduction in the phytoavailability of the lead in the *R. sphaeroides* treated soil.	[174]
*Rhodopseudomonas palustris* strain TN114 and PP803	Bacteria were selected from saline paddy fields and grown in glutamate acetate medium under photo-microaerobic conditions or dark aerobic conditions.	Liquid rice straw broth	Optimal growth conditions to enable the growth of PNSB were studied. ALA production and ability of these bacteria to reduce CH_4_ emissions were assessed.	Both strains grew well at 0.25 % NaCl. As the concentration of NaCl increased (0.35−6% NaCl), growth reduced. Both strains were able to produce ALA and reduce CH_4_ emissions under salt stress. Highest ALA production by strain TN114−25.63 μM whereas highest CH_4_ emissions by strain PP803 – 88.41%	[175]

### 3.4 Single-cell protein

Single-cell protein (SCP) is a form of crude or refined protein derived from algae, fungi and bacteria and used either for animal or human consumption [176,177]. While microalgae and cyanobacteria have widely been studied and used as an SCP, there are several advantages of using APB over algae. Apart from the economic advantages these bacteria are a good source of protein given that crude protein makes up 60–70 % of their total dry weight. They are also rich in essential amino acids, carotenoid pigments and vitamins, such as pantothenic acid, niacin, folic acid, vitamins E, B12, B2, B1 and B6, thus making them suitable for use as fish/animal feed [[178], [179], [180]]. Additionally, some APB have 20−25% of carbohydrate as their cell dry weight [180]. This high carbohydrate content makes the feed suitable for various warm water and freshwater fish [181]. APB, in addition to being used to promote the growth of fish and shrimps, can also enhance their immunity and protect them from diseases. Chumpol et al. [182] explored the potential of three strains of probiotic PNSB - *Rhodobacter sphaeroides* SS15, S3W10 and *Afifella marina* STW181 - in improving the survival of shrimp exposed to *Vibrio parahaemolyticus* (known to cause acute hepatopancreatic necrosis disease). They found that the PNSB probiotic mix was able to prevent shrimp disease, improve shrimp survival and growth and also maintain the quality of water used for shrimp cultivation. A similar study by Seangtumnor et al. [183] screened over 22 strains of PNSB and identified that *Rhodovulum sulfidophilum* PS342 was able to act as a biocontrol agent, by producing proteolytic enzymes to maintain water quality and anti-vibrio compounds to protect shrimp from bacterial diseases.

Among APB, PNSB is the most preferred group of bacteria for aquaculture-based studies because of its metabolic versatility and diverse applications range such as maintaining the quality of the cultivation water, as a probiotic and as a source of protein. Non-toxic organic wastewater including; soybean, dairy, peptone, cooking and palm oil effluent, wastewater from breweries and effluents from the sugar industry have been studied for the cultivation of phototrophic bacteria for SCP use [[184], [185], [186]]. PNSB such as *R. sphaeroides* Z08*, R. capsulata*, *Rhodopseudomonas gelatinosa*, *Rhodocyclus gelatinosus* R7 and *Rhodopseudomonas blastica,* were studied for SCP production and produced >50 % crude protein from wastewater substrates [[187], [188], [189]]. When compared to the FAO reference protein [190], protein from microalgae and cyanobacteria, the PNSB *R. capsulata* had the highest content of sulfur and essential amino acid content. High histidine residues were found in *Rhodospeudomonas rubrum*, while *Rhodospeudomonas tenue* was characterized by high valine and methionine content when the organisms were grown on clarified biogas plant effluent [188]. Table 4 highlights recent studies that have utilized anoxygenic phototrophic bacteria as animal/fish feed.

**Table 4:** Recent studies using anoxygenic phototrophic bacteria for single-cell protein production and resource recovery.

ORGANISM	CULTURE ORIGIN	TARGET	CULTURE CONDITIONS	PROTEIN CONTENT	ADDITIONAL OUTCOME	REFERENCE
*Rhodopseudomonas faecalis* PA2	Wastewater treatment pond	Animal feed additive	Anoxygenic growth in photobioreactor. 2000−4000 lux. Optimal growth was at light intensity of 4000 lx at 150 rpm	64.8 % protein. Essential amino acids (required for penaeid shrimp) accounted for 72.6 % of total protein content.	Carotenoid and bacteriochlorophyll produced	[191]
*Rhodopseudomonas*	Local pond	SCP	Dark aerobic conditions on biogas slurry with a salinity of 1.38 %	Protein content could be enhanced up to 90 % due to high ammonia and salinity of the biogas slurry	Upregulation of glutamate synthase and glutamine synthetase activity, that indicated enhanced glutamine and glutamate biosynthesis	[192]
*Rhodobacter sphaeroides* SS15, S3 W10, TKW17 and *Afifella marina* STW181	Isolated from shrimp ponds in south Thailand	Source of protein for *Litopenaeus vannamei* (white shrimp)	Cultured under basic isolation media with 1.5 % NaCl (*R. sphaeroides*) and glutamate acetate medium with 2% NaCl *(A. marina)* under photo-microaerobic conditions	Protein content for *R. sphaeroides* SS15 was the highest at 53.98 ± 0.08%, whereas for *A. marina* STW181 it was 49.06 ± 0.10 and the essential amino acid profile of these strains were higher than the essential amino acid requirements for shrimp	Among the different diet formulations tested, Diet 1 comprising of 1% commercial shrimp feed mixed with 0.5 % each of *R. sphaeroides* SS15 and *A. marina* STW181, was the most effective shrimp feed and it enhanced shrimp growth and survival.	[193]
*Rhodopseudomonas*	Local pond	Resource recovery	Artificial brewery wastewater cultivated in photobioreactors with natural light and microaerobic conditions	420.9 mg/g	The bacteria were rich in polysaccharides, carotenoids, bacteriochlorophyll and coenzyme Q10 (antioxidant)	[194]
Mixed purple phototrophic bacteria (PPB) inoculum	Enriched from wastewater	Fish meal replacement for *Lates calcarifer* (Asian sea bass)	Photo anaerobic bioreactor with Ormerod medium	> 57 %	Up to 66 % of fishmeal could be replaced with purple phototrophic bacteria and this did not cause any adverse effect on fish. Total amino acid present was highest when PPB replaced 66% and 100% of fishmeal.	[195]
PPB cultures initially dominated by *Allochromatium sp.* and *Rhodobacter sp.* but eventually by *Rhodovulum sp.*	Brisbane river water and mud sediments	Treatment of highly saline wastewater	Raw domestic wastewater was used as a substrate. Cultivation was carried out in a continuous infra-red photo anaerobic membrane reactor	0.62(0.21) gCP/gVS crude protein.	COD:N:P removal was 100:6:5:1.0 with 0.8 g COD/ gCOD_fed_	[196]
*Rhodopseudomonas faecalis* PA2	–	Animal feed	Cultures were initially grown on a glutamate malate medium in anaerobic light conditions. Wastewater from the sugar industry in Thailand was used for cultivation of the bacteria under microaerobic conditions	>50 %. All essential amino acids were present. Methionine, usually the limiting amino acid, was at higher concentrations than many other SCP sources.	Carotenoids production and COD reduction by 80 %	[197]

### 3.5 Antioxidants and pigments

Carotenoids are accessory pigments that are an essential component of the photosynthetic machinery and play a key role in harvesting light. Carotenoids support photosynthesis by actively absorbing light at a wavelength of 450−550 nm. Within this region, the solar radiation has the maximum intensity and can transfer solar energy to chlorophyll [198,199]. Carotenoid pigments stabilize light-harvesting complexes [31] and are vital for organisms, such as purple photosynthetic bacteria, living in environments with reduced availability of light [199]. Apart from acting as light-harvesting accessory pigments, carotenoids also protect and prevent the photosynthetic machinery from excess light-induced photodamage. When chlorophyll (Chl) and Bacteriochlorophyll (BChl) are induced by light to form triplet states, they then react with molecular oxygen to form singlet oxygen. These highly reactive species can cause oxidative damage to Chl, BChl, lipids, protein and DNA. Carotenoid pigments are able to quench this triplet state to protect the photosynthetic apparatus and the cell from photo-oxidative damage [198,200]. Different types of anoxygenic phototrophic bacteria have varying carotenoid pigments composition. The main pigments present are: *diaponeurosporene* in heliobacteria; *spheroidene*, *spirilloxanthin*, *lycopene,* and *rhodopsin* in purple phototrophic bacteria; *isorenieratene* for GSB and *α, β carotene* in green filamentous bacteria [22,31].

Since animals cannot synthesize carotenoids, they acquire them from the food they consume. The organic compound vitamin A is vital for the differentiation and development of various tissue across numerous species, but it cannot be synthesized *de novo* by animals and has to be obtained through the diet. The *β carotene* pigment that is found in plants is converted into retinal in vitro and is required for the biosynthesis of vitamin A [201,202]. Carotenoid pigments have widely been used as antioxidants and natural food coloring additives in the pharmaceutical, cosmetic and food industries [203]. Sunscreen and tanning products contain *β-carotene* and *lycopene* [204,205] for their coloring as well as antioxidant properties while *canthaxanthin* is often used to color fish and poultry [206,207].

The ease of APB culture and maintenance makes them suitable for carotenoids extraction and can be widely used at industrial level. A crucial factor that influences the production of carotenoids in these organisms is light. Zhou et al. [208] studied the effect of different types of light on carotenoid production in *Rhodopseudomonas* and reported that yellow light, at a wavelength of 595 nm, produced the highest amount of carotenoid compared to red, blue or white LED light. The intensity of light can also influence the amount and type of pigments produced. Muzziotti et al. [209] observed that *R. palustris* 42OL grown under low light (250 μmol photons m^−2^ s^-1^) and anaerobic/aerobic conditions produced large amounts of *lycopene* while these bacteria produced more polar carotenoids such as *rhodovibrin* and *rhodopin* when grown under high light intensities (1500 μmol photons m^−2^ s^-1^). A study by Saejung and Chewapat [191] using *Rhodopseudomonas faecalis* PA2, found that among the different light intensities tested the highest carotenoid yield and production was seen at 4000 lx and at intensities greater than this, carotenoid production decreased. This was because carotenoid pigments are degraded at high light intensities to protect cells from photo-damage [209]. Therefore, at low light conditions, there is an increase in carotenoid production to efficiently capture solar energy required for its growth and metabolism. Salinity, aeration, additives and biostimulants can also influence carotenoid production. Table 5 provides the main highlights of various studies on the assessment of factors enhancing carotenoid production by PNSB.

**Table 5:** Recent studies using purple non-sulfur bacteria for carotenoid production.

FACTOR	SUBSTRATE	CONDITIONS	EFFECT	ORGANISM	REFERENCE
Light	Artificial sugar wastewater.	Photo-anaerobic conditions with 4 light: dark cycles- 3:3 h, 6:6 h, 12:12 h and 24:24h	Carotenoids were stable under different cycles which indicated that cycling of light was vital for carotenoid production. The maximum carotenoid output was during the 12 h dark:12 h light cycle (∼1.4 mg/L)	Mainly *Rhodopseudomonas*	[222]
Substrate – different agro-industrial waste	Soy, coconut and cassava meal	Photo-anaerobic conditions	Carotenoid productivity was the highest with cultivation in soybean meal medium 71.25 mg/L (over a period of 10 days). High soybean meal concentrations contains large amounts of protein which affects pH and in turn affects the carotenoid formation, due to reduced bacterial growth [223]. Hence soybean meal at 50% concentration was the ideal substrate for carotenoid production. Carotenoids such as *lycopene 1,2-dihydrolycopene, cis-1,2-dihydrolycopene* and *1,2-dihy- dro-3,4-dedihydrolycopene* were synthesized.	*Rhodopseudomonas faecalis* PA2	[136]
Additives -yeast	Artificial brewery wastewater	Natural light and micro-aerobic conditions without aeration	Yeast extract at 400 mg/L was the best for the growth of the bacteria, and at this concentration of yeast the carotenoid production was 2.53 mg/g. The addition of 400 mg/L yeast increased dehydrogenase activity, which was indicative of biomass growth [224].	*Rhodopseudomonas*	[194]
High salinity	Synthetic highly saline wastewater	Bioreactor based photo-anaerobic cultivation, under 20, 50 and 100 g/L NaCl concentrations.	Carotenoid production was optimal (3 mg/L) when 50 g/L NaCl was used, this was higher than the control. However, when the concentration of NaCl was further increased to 100 g/L, carotenoid production was the lowest. A similar trend was observed in the case of bacteriochlorophyll concentrations. Dehydrogenase activity was the highest at 50 g/L NaCl, as a consequence of high biomass yield (over 144 h) and carotenoid production.	*Rhodopseudomonas strain* Z16	[225]
Light intensity and agitation speed	Domestic wastewater sourced from a pond	Cultivation in bioreactors under photo-anaerobic conditions. 4 different light intensities – 2000−5000 lx, and 3 different agitation speeds -150, 300 and 600 rpm	The highest carotenoid yield was 7.2 mg/g, while the highest carotenoid production rate was 74.3 mg/L and carotenoid productivity was 40.9 mg/L. Among the different light intensities tested, 4000 lx showed the highest carotenoid yield, production and productivity. As the light intensity increased, the photosynthetic rate increased resulting in high biomass productivity and production. However, when the intensity was >4000 lx, biomass and carotenoid decreased as a result of photoinhibition. In the case of different agitation speeds, a speed of 150 rpm resulted in maximum biomass productivity and production and highest carotenoid yield, production and productivity. Agitation of the culture led to an increase in mass transfer rates and decreased light transfer limitations.	*Rhodopseudomonas faecalis* PA2	[191]
Dissolved oxygen (DO)	Artificial sugar wastewater	Photo-bioreactors illuminated by natural light. Agitators were used to set different dissolved oxygen (DO) concentrations - <0.5, 0.5–1, 1–2, 2–4, 4–8 mg/L.	DO of <0.5 mg/L showed highest carotenoid production over a period of 96 h, ∼ 1.7 mg/L, and could be attributed to the photophosphorylation metabolic pathway adopted by the organism [226] (oxidative phosphorylation at DO concentrations >0.5 mg/L). Dehydrogenase activity was also highest at DO of <0.5 mg/L.	*Rhodopseudomonas*	[227]
Biostimulant *Bacillus thuringiensis/ cereus* L2	Synthetic peptone wastewater	Cultivation in photo-bioreactors under micro-aerobic conditions. Different volumes of biostimulants were used- 0, 5, 10, 20, 40 and 80 μL	Co-culture of *R. sphaeroides strain* (ATCC17023) with the addition of 40 μL of L2 had the highest carotenoid yield - 3.24 mg/g biomass and a concentration of 11.8 mg/L. Intracellular peroxidase activity (encoded by RSP_3419 gene in *R. sphaeroides* and degrades intracellular carotenoid content) was the lowest when 40 μL L2 was added to the culture. Therefore, the addition of L2 at an optimal volume of 40 μL inhibited peroxidase activity and thus increased carotenoid yield.	*Rhodobacter sphaeroides strain* (ATCC17023)	[228]
Additives – Magnesium ion (Mg^2+^)	Artificial sugar wastewater	Cultivation in bioreactors under photo-micro aerobic conditions. Different concentration of Mg^2+^ were added - 0, 1, 5, 10, 15 and 20 mmol/L	The highest carotenoid yield of 4.83 ± 0.14 mg/g was obtained when 15 mmol/L Mg^2+^ was added. The same concentration of Mg^2+^ upregulated the expression of the *crtBDA* genes and increased carotenoid yield (*CRT* genes are responsible for the biosynthesis of carotenoids [229])	*Rhodobacter sphaeroides strain* (ATCC17023)	[230]
Additives – magnesium ion (Mg^2+^)	Soybean wastewater	Bioreactor based photo-anaerobic cultivation with. different concentration of Mg^2+^ - 5, 10 and 15 mg/L	Addition of 10 mg/L of Mg^2+^ to the cultures resulted in highest bacteriochlorophyll content and ATP production - ∼11.5 μg/mg and 35 μg/mg respectively. Mg^2+^ function as active sites of enzymes and pigments, it plays a vital role in capturing solar energy and converting it to electrons. [231]. Thus, the addition of an optimal dose of the additive improved bacteria growth and increased bacteriochlorophyll content which triggered an increase in ATP production (bacteriochlorophyll content regulates ATP/ energy production from photosynthesis)	*Rhodobacter sphaeroides* Z08	[232]
Type of light	Sugar wastewater	Cultivation was in photo bioreactor with DO concentration <0.5 mg/L. Different light sources were used - red LED (650 nm, 18 W), a yellow LED 595 nm, 18 W), blue LED (470 nm, 18 W), white LED (9 W), and incandescent lamp (400–780 nm, 80 W).	Compared to incandescent lamps, carotenoid and bacteriochlorophyll production were highest for the cultures exposed to LED lights. Highest carotenoid and bacteriochlorophyll production were reported for the yellow LED cultures ∼950 mg/L and 900 mg/L respectively. Since carotenoids strongly absorb light in the range of 450−550 nm and also the visible part of the spectrum, it was synthesized at a higher concentration when compared to bacteriochlorophyll.	*Rhodopseudomonas*	[208]

Ubiquinone is commonly referred to as coenzyme Q (CoQ), and is produced in animals and most bacteria. The most common type pf CoQ present in humans is CoQ_10_, and since they can sequester electrons, they act as antioxidants by protecting lipoproteins or lipids from oxidation [210]. They also play a role in the oxidation of sulfide [211] and regulate mitochondrial permeability transition pore and the transfer of protons, like Ca^2+^, across biological membranes [212]. A deficiency in CoQ_10_ in humans leads to several central nervous system, metabolic and cardiovascular disorders [[213], [214], [215]]. Although CoQ_10_ can be chemically synthesized; lack of stereoselectivity, high substrate cost, low yield and high chemical waste are some of the major drawbacks associated with this mode of production [216,217]. Therefore microbial biosynthesis of this conenzyme is the best alternative. There are several studies that have utilized APB for the synthesis of CoQ_10_. An et al. [218] evaluated the antioxidant activities of *Rhodobacter sphaeroides* and demonstrated the ability of these bacteria to alleviate oxidative stress induced in Caco-2 cells. Zhang et al. [219] revealed that phosphate limitations could improve the biosynthetic efficiency of CoQ_10_ production in *R. sphaeroides* strain HY01. Yajima et al. [220] have a patented process for culturing reduced CoQ_10_ from a mixed group of organisms that include PNSB such as *Rhodopseudomonas palustris* JCM 2524 90 6 and *Rhodobacter capsulatus* SB 1003 95 6. Since *R. sphaeroides* is a native producer of this antioxidant, this PSNB is the ideal host for CoQ_10_ on an industrial scale [221].

## 4 Metabolic engineering of anoxygenic phototrophic bacteria for bioremediation and bioproduction

Synthetic biology applies the principles of engineering to design new biological systems or redesign existing systems for varied medical and environmental applications, and for the production of valuable chemical compounds. An important component of synthetic biology is identifying a compliant host ‘chassis’ with the desired phenotypic traits required for the end application. Thus far, only ten engineered microbes have been well studied for industrial applications [233]. By isolating and studying organisms occurring as a part of the natural biodiversity with distinct metabolic networks that can accommodate specific chemical reactions, more host organisms can be discovered [233]. O’Neill et al. [234] used a yeast-bacteria hybrid cloning system to alter photosynthetic metabolism in *Chlamydomonas reinhardtii*. Engineered *Pseudomonas putida,* which thrive in toxic environments, has been well studied for its bioremediation potential and can be used as a host organism for several applications within the chemical industry [[235], [236], [237]].

Like most phototrophs, the efficient utilization of light by APB is vital for its growth and metabolism. Most photosynthetic proteins are limited in their use of solar energy due to specific light-harvesting pigments that absorb light at particular wavelengths. To improve photosynthetic efficiency, Liu et al. [238] designed a self-assembling photoprotein chimera using the reaction center (RC) of *R. sphaeroides* and the light-harvesting component II (LH2) from *Arabidopsis thaliana.* This engineered chimera enabled polychromatic harvesting and conversion of solar energy that extended through the near-UV, visible and near-IR region. Swainsbury et al. [239] improved the light harvesting efficiency of *R. sphaeroides* also by modifying the LH2 complex. They modified the B800 (BChl absorbing at 800 nm) binding site specificity, through the addition of a hydrogen bond to the 3-acetyl group Arg_−10_ of the LH2 β polypeptide, to enable equal binding of Chl *a* and BChl *a*. This improved the light-harvesting ability of LH2 at wavelengths within the red-gap, while retaining light-harvesting efficiency.

Sesquiterpenoids belong to a class of diverse organic products, derived from farnesyl pyrophosphate (FPP), that are used in the food, agriculture, cosmetic and pharma industry [240]. They are synthesized either via the mevalonate (MVA) pathway [241] or the 1 deoxy-d-xylulose 5-phosphate (DXP) pathway [242]. Bacteria from the genus *Rhodobacter* have a large intracytoplasmic membrane system that accommodates components of the photosynthetic machinery [243,244], metabolites and membrane-embedded enzymes [240]. Troost et al. [240] used *R. capsulatus* as a host for the production of two plant sesquiterpenoids, patchoulol and valencene. *FPP synthase* (*IspA*), rate-limiting enzymes *DxS synthase* and isopentenyl pyrophosphate isomerase (*IPP isomaerase),* and selective enzymes from the MVA pathway (derived from *Paracoccus zeaxanthinifaciens)* were co-expressed in *Rhodobacter* to increase the production of recombinant terpenoid. Co-expression of the DXP/MVA genes were controlled by the P*_nif_* promoter. An increase in patchoulol and valencene titers were reported and this study validated the use of the purple bacteria, *R. capsulatus,* as a suitable chassis for the production of sesquiterpenoids. Giraud et al. [245] redirected the synthesis of *spirilloxanthin* towards the production of *β-carotene* and *canthaxanthin,* by replacing the *crtCD* genes (involved in the synthesis of *spirlloxanthin* from *lycopene)* with *crtY* (involved in the cyclization of lycopene to β-carotene) and *crtW* genes (oxygenates β-carotene) that were derived from *Bradyrhizobium* ORS278.

Higuchi-Takeuchi et al. [246] evaluated two methods for the transformation of two marine photosynthetic purple bacteria, *Rhodovulum sulfidophilum* and *Roseospira marina*, to develop ready-to-use host cells. Following treatment of the cells with calcium chloride and introducing plasmid DNA into the competent cells, they found that heat shock treatment improved transformation efficiencies compared to use of cell-penetrating peptides. Developing a suitable ‘chassis’ requires the selection of organisms with the right phenotypic traits and the right genome editing tools. SpCas9 from *Streptococcus pyogenes* is the Cas9 RNA-guided DNA-endonuclease from the type II CRISPR-Cas system, and has been widely studied as a genome editing tool for both prokaryotes and eukaryotes. Mougiakos et al. [247] combined the SpCas9 DNA targeting system with homologous recombination templates from *R. sphaeroides* to develop an efficient genome editing tool. This tool was implemented in the genome of *R. sphaeroides* to delete the acetyl-CoA reductase genes – *phaB* and *phbB.* The authors were able to identify the dominant PHB production pathway and engineer mutant stains with reduced PHB production, thus validating the efficiency of this system for gene knock-outs/knock-ins and single nucleotide substitution. A similar system can also be utilized to improve photosynthetic efficiency in APB, increase PHA production by inactivating PHA depolymerase, and enhance the biosynthesis of terpenes and other value-added compounds in APB.

## 5 Summary

### 5.1 Outlook

Anoxygenic phototrophic bacteria can potentially be used for a wide array of applications including wastewater treatment, bioremediation, recycling energy (Photo-BES technology) and production of value-added substances (pigments, PHA, protein and biofertilizers) [248]. Cheap growth substrates, low maintenance cost and absence of associated toxins give them an advantage over conventional algae/cyanobacteria based technology [6,249]. Among the APBs, the PPB, due to their metabolic versatility, have been well studied and characterized for resource recovery applications and wastewater treatment. Although APB based technology can potentially be used for an array of environmental biotechnology applications there are several limitations associated with this technology.

### 5.2 Limitations

Only a few APB, notably PNSB, have been characterized and studied for wastewater-based resource recovery and they may not have a competitive advantage over other heterotrophic bacteria when used in open systems [248]. From a financial point of view, the capital expenditure cost and operational expenditure costs associated with photobioreactors can also prove to be bottlenecks to this emerging technology. Complicated reactor designs resulting in the need to optimize light and electrical efficiency; expensive materials associated with lighting, membranes and separators; low market price for specific products; biofouling and lack of proper legislation governing the commercial use of APB are some of the common limitations that need to be addressed to ensure the viability of this promising technology [248,250].

### 5.3 Future directions

Alloul et al. [251] estimated the cost of production of PNSB using a two-stage approach and cultivated using brewery wastewater to be €10/kg dry weight (0.5 days SRT), which is ∼20 % cheaper than the cost of microalgae production [251,252]. Sakarika et al. [253] compared the rough estimates of PNSB products to the market prices and identified that compared to the commercial product, the cost of ALA from PNSB was more economical. Depending on the purity grade, the price for 5-ALA is over $1300/5 g [254], while carotenoid pigment and bacteriochlorophyll are priced between $350-$7500/kg [255] and over $1700/5 g [256] respectively. SCP and PHA have the lowest market price at $1−20/kg [257] and $1−7/kg [258] respectively. The choice of carbon substrate, the type of APB used and the configuration of the bioreactor can significantly affect the cost and can determine the sustainability of the technology. Most likely, the economic value will come from focusing on high-value products, though systems that can simultaneously generate and recover other resources will be advantageous.

Resource recovery studies should focus on utilizing low-cost non-toxic organic substrates like wastewater from the soybean meal, olive mill, sugar and molasses industry [135,136]. The upstream processing of substrate can be optimized in several ways to lower overall cost [250]. This can be achieved by concentrating the carbon source; and implementing pre-treatment steps such as ultrafiltration to remove proteinaceous retentate [259] and enzymatic/chemical hydrolysis to break long chain sugar to simple sugars [260]. Charcoal and similar material can be used to adsorb toxic by-products [261] and consequently increase yield, reduce production costs, enhance bioconversion efficiencies, and decrease both operational expenditure and capital expenditure during scaling up operations [138]. For photo-bioreactors relying on natural light, the exploration of various light collection systems such as fiber optic solar collectors as a means of concentrating solar energy and making it readily available for phototrophic organisms should be explored. The use of IR filters to capture IR wavelengths from sunlight should be studied, particularly for large scale operations.

Selection and exploration of different APB, characterizing their metabolic and biosynthetic pathways, is key to improving wastewater treatment and maximizing the production of high- value products. A better comprehension of the ecology of APB using RNA and DNA based metagenomics, environmental proteomics and molecular fingerprinting can greatly help in identifying organisms and substrates that are best suited for the production of particular high-value substances [8]. By mapping the genes that play a key role in specific biosynthetic pathways, synthetic biology can be used to enhance the production of these high-value resources [239,240,245].

APB based technology has the potential to be utilized for a wide variety of applications in the food, agriculture, aquaculture, bioremediation and pharmaceutical industry. A holistic approach to the sustainable production of APB based value-added substances needs to be adopted. Collaborative efforts from various scientific fields, as well as academia and industry, are vital for the commercialization and scaling up of this technology.

## Declaration of Competing Interest

The authors declare that they have no known competing financial interests or personal relationships that could have appeared to influence the work reported in this paper.
